# Kinematic differences in the in-match serve between top-ranked and lower-ranked Women's Tennis Association players

**DOI:** 10.3389/fspor.2026.1787475

**Published:** 2026-04-23

**Authors:** Binxi Gao, Haiyang Hu, Yuduo Liu, Huimeng Chen, Xianglin Wan

**Affiliations:** 1 Biomechanics Laboratory, School of Sport Science, Beijing Sport University, Beijing, China; 2 Physical Education College, Jiujiang University, Jiujiang, China

**Keywords:** biomechanics, projection angle, tennis serve, three-dimensional kinematic, women's tennis

## Abstract

**Background:**

Although high serve velocity is recognized as a decisive factor in women's tennis, the specific in-match kinematic variations that distinguish top-ranked players from lower-ranked competitors remain under-investigated.

**Methods:**

The videos of the first serve were collected at competition venues from seven top-ranked players (ranked within the top 40 as of September 2019) and ten lower-ranked players (ranked outside the top 100). Joint angles were calculated using three-dimensional projection angle method based on the kinematic data obtained from FM3DMotion software. Independent-samples t-test and Mann–Whitney U test were employed to compare the kinematic differences between the two groups.

**Results:**

Ball velocity, elbow angle at maximum knee flexion, elbow angle at the racket's lowest position, and peak elbow angular velocity during the cocking phase were greater in top-ranked players (*p* < 0.05). The center of mass to racket side foot distance, shoulder external rotation angle at maximum knee flexion, occurrence time of hip peak linear velocity, and ranges of trunk flexion/extension and lateral flexion during the acceleration phase were greater in lower-ranked players (*p* < 0.05).

**Conclusions:**

Kinematics such as an elevated upper arm and open elbow at MKF, a rapid elbow flexion during cocking phase, and dominant trunk rotation are characteristic of elite players. These patterns may serve as potential technical targets for developing players aiming to optimize serve velocity.

## 1 Introduction

The tennis serve is one of the most important actions in the match, as it initiates the rally. A high-quality serve provides the server with a significant advantage in winning point (1). According to the 2025 Women's Tennis Association (WTA) statistics, the percentage of first serve points won by the top ten players is over 65% (2). Among the various factors associated with tennis serve, ball velocity is one of the most decisive factors influencing match performance (3), with a particularly pronounced impact on the success of female players (4). Therefore, enhancing serve velocity is a key strategy for improving competitive performance of female players. Understanding the biomechanical mechanisms of generating high velocity is a prerequisite for optimizing serve technique.

As an upper extremity whip-like movement, the tennis serve generates extremely high velocity at the distal end, wherein the effective coordination of various body segments is essential for maximizing the whipping effect (5). Previous studies indicated that the power of serve primarily derives from the increased activity of the pronator teres and triceps brachii (6). Both shoulder internal rotation and elbow extension significantly impact serve velocity, with the former contributing approximately 41.1% to the racket head speed (7, 8). Similarly, increasing elbow extension capacity during swing leads to a faster serve (9, 10). Beyond the racket arm, sufficient trunk rotation (3), faster knee extension speed (11), and a larger range of knee flexion have also been shown to correlate closely with higher ball velocity (12, 13). However, due to constraints in data acquisition during actual matches, previous studies have been primarily conducted in training or laboratory settings (12, 14, 15). Such motion patterns may differ from the serve techniques employed during competition due to factors such as fatigue, tactical considerations, and psychological pressure (16). In this sense, in-match analysis can provide field-based perspective for the improvement of serve technique.

Additionally, the aforementioned conclusions primarily apply to collegiate and average-level professional players. The serve kinematics of world's top female players remain unclear. Notably, serve velocity is positively associated with competitive level and ranking (17, 18). This suggests that world-top players’ movement patterns may more effectively utilize biomechanical principles to maximize ball velocity. Thus, analyzing serve technique of top players and comparing them with those of lower-level players, will provide critical insights conducive to the targeted design of scientific training interventions.

Given the critical role of the serve in women's tennis and the lack of biomechanical analyses derived from real match play, the current study aimed to find the technical disparities of serve between top-ranked and lower-ranked WTA players and provide theoretical references for the improvement of scientific training. It was hypothesized that serve velocity of top-ranked players is higher than that of lower-ranked players. And it was also hypothesized that players of different WTA rankings exhibited different kinematics during various serve phases.

## 2 Materials and methods

### 2.1 Participants

This study included seven top-ranked female professional tennis players (hereinafter referred to as top-ranked players, age: 27.1 ± 3.6 years; height: 1.72 ± 0.06 m; mass: 63.0 ± 4.5 kg; world ranking: 16 ± 11), ranked within the top 40 of the WTA world rankings as of September 2019. These players participated in the 2019 WTA Open tournaments in Guangzhou and Wuhan. Additionally, ten female professional tennis players (hereinafter referred to as lower-ranked players, age: 21.3 ± 2.1 years; height: 1.71 ± 0.05 m; mass: 61.4 ± 2.9 kg; world ranking: 451 ± 228), ranked outside the top 100 in the WTA world rankings, participated in the 2020 China Open Year-end Finals and the 2024 Beihai national team training camp scrimmage. All players in this study were right-handed and employed foot-up serve.

### 2.2 Data collection

**Figure 1:**
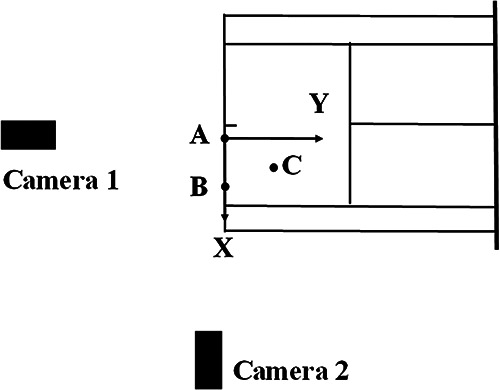
Camera's position and global coordinates system diagram.

The videos of serve during competitions were recorded using two high-speed cameras (FDR-AX700, SONY, Japan, Video resolution: 1080 p; Frame rate: 120 Hz; Shutter speed: 1/1000 s). The cameras were positioned 15 m behind and 15 m to the right of the player, respectively. A PEAK radial three-dimensional calibration frame with 32 control points was used to calibrate the filming space. Additionally, three ground markers (labeled A, B, and C) were employed to establish the global coordinate system. The *X*-axis points to the right side of the court, the *Y*-axis extends forward along the long axis of the tennis court, and the Z-axis points vertically upward. Point A serves as the origin, point B is located on the *X*-axis, and point C lies within the X-Y plane (Figure 1). The serve velocity was obtained from the official tournament statistics.

### 2.3 Data processing

For each player, first serves directed to the inside corner of the advantage court were recorded. The trial with the highest ball velocity was selected for analysis, as it was considered to represent the player's maximal performance. Twenty-three analysis points on the body were identified, including head, chin, neck, xiphoid, navel, and both sides of the shoulders, elbows, wrists, hands, hips, knees, ankles, heels and toes. Additionally, the ball, racket head, racket handle, and inner side of the racket were also identified. All points were manually digitized frame-by-frame from two synchronized camera views using the FM3DMotion system (Version 2.2.5.228, Fast Move Technology, Dalian, China). The three-dimensional coordinates were then calculated and filtered using a Butterworth recursive low-pass digital filter with a cut-off frequency of 10 Hz (19).

The coordinates of the center of mass (COM) were calculated using the DeLeva-adjusted Zatsiorsky-Seluyanovs body segmental inertia parameters (20). Joint angles were defined and calculated using the three-dimensional projection angle method (21) (see Supplementary Material 1 for the definition of the model).

Regarding ball trajectory, the ball release height was defined as the vertical distance from the ball to the ground at the release moment. The maximum ball ascent height was defined as the vertical distance from the ball's highest point in the air to the ground. The impact height is defined as the vertical distance between the ball and the ground at the moment of impact. The drop height was defined as the difference between ball maximum height and impact height. Additionally, COM to foot distance was defined as the distance between the projection of the COM and the foot vector on the ground. The joint angular velocity is defined as the change in joint angle per unit time, expressed in rad·s^−1^. The peak linear velocity occurrence time was defined as the time interval between the moment when the peak linear velocity occurs and the moment of impact. The range of motion (ROM) was defined as the difference between the maximum and minimum joint angles during serve.

**Figure 2:**
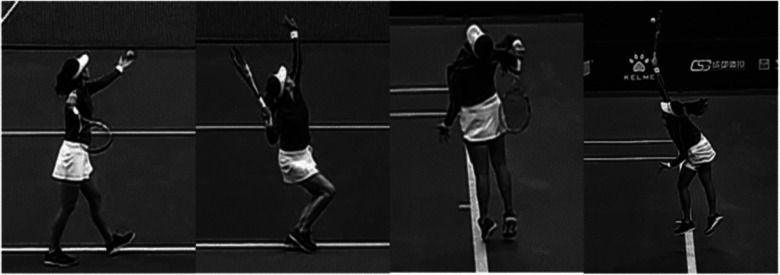
The key events of tennis serve. From left to right, these events are BR, MKF, RLP and impact.

The serve motion was analyzed based on four events: ball release (BR), maximum knee flexion (MKF), racket lowest position (RLP), and impact (Figure 2). BR is defined as the first frame when the ball separates from player's hand. MKF is defined as the moment of maximum flexion of racket side knee. RLP is defined as the lowest point of the racket head trajectory when the racket is positioned behind the body. Impact is defined as the initial contact between the ball and the racket. These events divided the serve into three phases: the loading phase (from BR to MKF), the cocking phase (from MKF to RLP), and the acceleration phase (from RLP to impact) (22, 23).

### 2.4 Statistical analysis

For continuous variables, the normality of the data was assessed using the Shapiro–Wilk test. If the data followed a normal distribution and exhibited homogeneity of the variances, intergroup comparisons were conducted using independent-samples t-test, and the data were expressed as mean ± standard deviation (SD). Otherwise, the Mann–Whitney U-test was employed, and the data were expressed as median and interquartile range (IQR). Cohen's d was provided as a measure of effect size for the independent-samples t-test, with the following classifications: small effect (0.2 ≤ *d* < 0.5), medium effect (0.5 ≤ *d* < 0.8), and large effect (*d* ≥ 0.8) (24). Additionally, the rank-biserial correlation was calculated as a measure of effect size for the Mann–Whitney U test, where the range of |*r*| is from 0 to 1. A value of |*r*| closer to 1 indicates a greater difference between the groups (25). Statistical significance was defined as the probability of a Type I error not exceeding 0.05. All statistical analyses were performed using SPSS 26.0 (IBM Cooperation, Armonk, NY, USA).

## 3 Results

Ball velocity (lower-ranked players: 116.9 ± 24.0 km·h^−1^, top-ranked players: 164.0 ± 9.6 km·h^−1^, t = 4.892, *p* < 0.001) was slower in the lower-ranked player. Racket side COM-foot distance at MKF (18.4 ± 6.3 cm, 12.8 ± 3.0 cm, t = 2.191, *p* = 0.045), and occurrence time of hip peak linear velocity (median [IQR] = 116.7 [2.1] ms, median [IQR] = 108.3 [8.3] ms, U = 8.0, z = 2.736, *p* = 0.006) were greater in the lower-ranked players than in the top-ranked players. Other parameters showed no significant difference between the two groups (*p* > 0.05) (Tables 1, 2).

**Table 1:** Ball kinematics and COM-foot distance.

Variables	Lower-ranked	Top-ranked	*p*	ES
Ball velocity (km·h^−1^)	116.9 ± 24.0	164.0 ± 9.6	<0.001^a^	2.577
Ball release height (m)	1.72 ± 0.11	1.75 ± 0.14	0.635	0.238
Ball maximum height (m)	3.56 ± 0.30	3.65 ± 0.26	0.514	0.321
Impact height (m)	2.68 ± 0.09	2.71 ± 0.07	0.541	0.372
Drop height (m)	0.88 ± 0.32	0.94 ± 0.23	0.637	0.215
Ball X velocity at BR (m·s^−1^)	0.2 ± 0.1	0.2 ± 0.1	0.912	<0.001
Ball Y velocity at BR (m·s^−1^)	0.3 ± 0.3	0.4 ± 0.3	0.658	0.239
Ball Z velocity at BR (m·s^−1^)	6.0 ± 0.6	6.2 ± 0.5	0.479	0.365
COM to racket side foot distance at MKF (cm)	18.4 ± 6.3	12.8 ± 3.0	0.045^a^	1.135
COM to non-racket side foot distance at MKF (cm)	6.2 ± 5.3	5.4 ± 2.9	0.716	0.187

^a^ Indicate statistical significance.

**Table 2:** The peak linear velocities and their occurrence times of each segment during acceleration phase.

Variables	Lower-ranked	Top-ranked	*p*	ES
Peak linear velocity (m·s^−1^)				
Racket head	29.6 ± 3.0	27.9 ± 1.8	0.195	0.687
Wrist	10.7 ± 0.7	10.5 ± 0.6	0.536	0.307
Elbow	7.9 ± 0.7	7.9 ± 0.6	0.962	<0.001
Shoulder	4.2 ± 0.6	4.2 ± 0.4	0.886	<0.001
Hip	2.5 ± 0.3	2.4 ± 0.2	0.417	0.392
Knee	1.9 ± 0.4	2.1 ± 0.3	0.315	0.566
Ankle	2.1 ± 0.5	2.0 ± 0.4	0.739	0.221
Occurrence time of peak linear velocity (ms)				
Racket head	4.2 (16.7)	8.3 (8.3)	0.836	0.057
Wrist	41.7 (16.7)	41.7 (8.3)	0.160	0.400
Elbow	89.2 ± 20.4	78.6 ± 19.8	0.459	0.527
Shoulder	90.0 ± 20.0	83.3 ± 22.6	0.489	0.314
Hip	116.7 (2.1)	108.3 (8.3)	0.006^a^	0.771
Knee	116.7 (20.9)	108.3 (25.0)	0.175	0.386
Ankle	81.6 ± 46.1	89.3 ± 22.4	0.961	0.212

IQR for non-normal distribution variables is represented in parentheses.

^a^ Indicate statistical significance.

**Figure 3:**
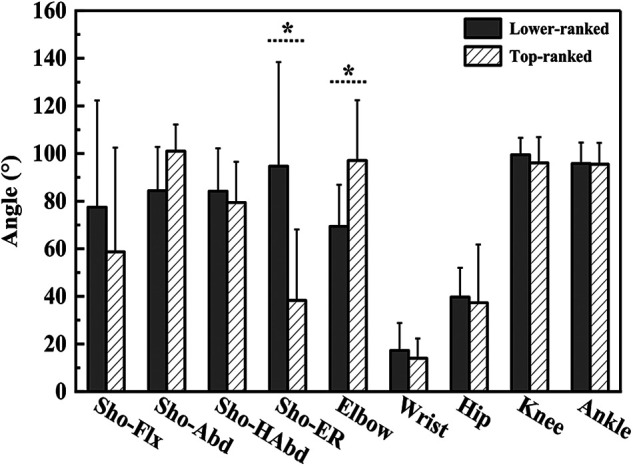
The joint angles of upper and lower extremity on racket side at the MKF. Sho, shoulder; Flx, flexion; Abd, Abduction; HAbd, horizontal abduction; ER, external rotation. * Indicate differences between groups *p* < 0.05.

**Figure 4:**
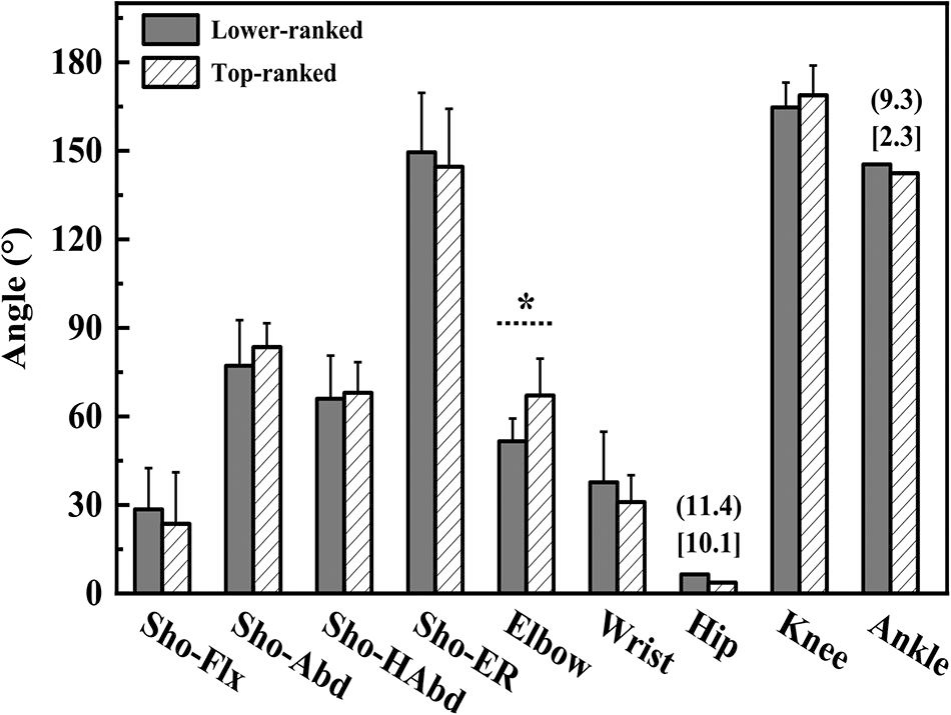
The joint angles of upper and lower extremity on racket side at the RLP. Sho, shoulder; Flx, flexion; Abd, Abduction; HAbd, horizontal abduction; ER, external rotation. * Indicate differences between groups *p* < 0.05. IQR for variables of lower-ranked players is represented in parenthesis. IQR for variables of top-ranked players is represented in brackets.

**Figure 5:**
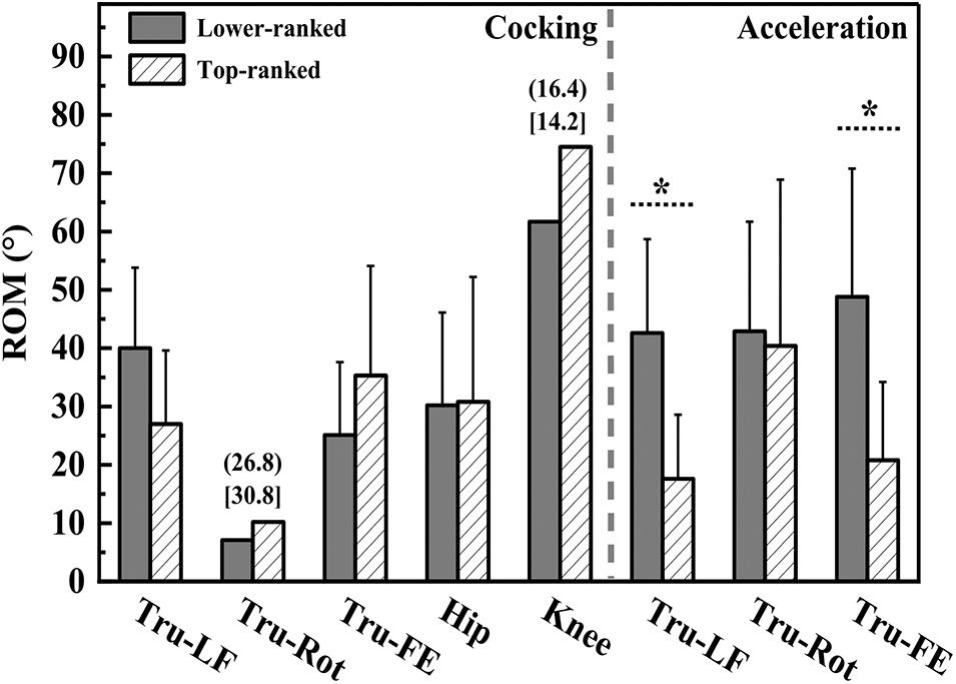
The ROM of trunk, hip, and knee during cocking phase and the ROM of trunk during acceleration phases. Tru, trunk; LF, lateral flexion; Rot, rotation; FE, flexion and extension. *Indicate differences between groups *p* < 0.05. IQR for variables of lower-ranked players is represented in parentheses. IQR for variables of top-ranked players is represented in brackets.

**Figure 6:**
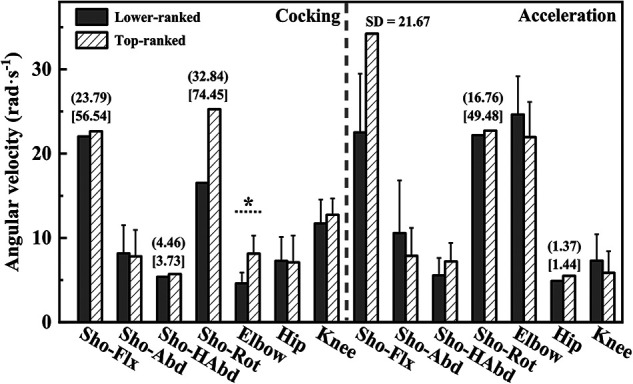
The peak angular velocities of upper and lower extremity during cocking and acceleration phases. Sho, shoulder Flx, flexion; Abd, abduction; HAbd, horizontal abduction; ER, external rotation. *Indicate differences between groups *p* < 0.05. IQR for variables of lower-ranked players is represented in parentheses. IQR for variables of top-ranked players is represented in brackets.

Shoulder external rotation angle at MKF (94.7 ± 43.7°, 38.3 ± 29.8°, t = 2.956, *p* = 0.010, Cohen's *d* = 1.508), trunk flexion and extension ROM during acceleration phase (48.8 ± 22.0°, 20.8 ± 13.4°, t = 2.977, *p* = 0.009, Cohen's *d* = 1.537), and trunk lateral flexion ROM during acceleration phase (42.6 ± 16.1°, 17.6 ± 11.0°, t = 3.556, *p* = 0.003, Cohen's *d* = 1.813) were greater in the lower-ranked players than in the top-ranked players. Elbow angle at MKF (69.4 ± 17.5°, 97.1 ± 25.3°, t = 2.685, *p* = 0.017, Cohen's *d* = 1.273), elbow angle at RLP (51.6 ± 7.7°, 67.1 ± 12.5°, t = 3.180, *p* = 0.006, Cohen's *d* = 1.493), and elbow peak angular velocity during cocking phase (4.60 ± 1.29 rad·s^−1^, 8.13 ± 2.14 rad·s^−1^, t = 4.262, *p* < 0.001, Cohen's *d* = 1.998) were smaller in the lower-ranked players than in the top-ranked players. Other parameters showed no significant difference between the two groups (*p* > 0.05) (Figures 3–6).

## 4 Discussion

The purpose of this study was to identify the technical disparities of serve between top-ranked and lower-ranked WTA players. The findings supported the hypothesis that top-ranked players generated approximately 28.7% higher ball velocity than lower-ranked players. The finding also supported the hypothesis that players of the two levels exhibited different kinematics during various serve phases. Overall, top-ranked players exhibited a more relaxed upper extremity on the racket side, faster elbow flexion angular velocity, and greater reliance on trunk rotation for creating racket lag. These technical differences might contribute to the observed ball velocity disparity, suggesting that the serve mechanics of top-ranked players are more effective in generating higher ball velocity.

Clarifying the differences in movements is a prerequisite for improving techniques. The current study found that at MKF, the upper arm position of lower-ranked players is slightly below the shoulder extension line, with the elbow tightly closed forming an acute angle of approximately 70°, and the forearm closer to the frontal plane of body due to a larger shoulder external rotation angle of 95°, which is roughly 2.5 times greater than that of top-ranked players. In contrast, top-ranked players have a more extended arm, with the upper arm above the shoulder extension line, elbow forming nearly a right angle at 97°, and forearm directed obliquely upwards towards the horizontal plane. Previous studies indicated that the biceps brachii muscle exhibits maximal strength at an elbow joint angle of approximately 56°, with strength gradually diminishing as the angle increases (26). While lower-ranked players demonstrate excessive tension in the elbow, they operate at a more optimal flexion angle for force generation, which theoretically should lead to a greater elbow flexion angular velocity. However, these players exhibit a lower peak elbow flexion angular velocity of only 4.60 rad·s^−1^, compared to 8.13 rad·s^−1^ in top-ranked players. This finding suggests that the tightly closed elbow position and excessive tension may create a less efficient posture, restricting their ability to rapidly accelerate the arm during this phase. Additionally, a significant difference was observed in the elbow angle at RLP between two groups, where lower-ranked players exhibited a tighter angle of 52° compared to 67° in top-ranked players. This excessive elbow flexion in lower-ranked players may adversely affect subsequent elbow extension during the swing. For the triceps, an elbow angle of approximately 84° is optimal for triceps force generation (26). The elbow position of lower-ranked player appears less favorable. This suboptimal position likely restricts their effective range of motion, potentially limiting the rapid elbow extension acceleration required before impact, thereby leading to a reduction in ball velocity. This likely limits their ability to generate maximal elbow extension strength, potentially leading to a reduction in ball velocity.

The findings showed that top-ranked players exhibited a greater peak elbow flexion angular velocity during the cocking phase. As a joint located near the end of the kinetic chain, the elbow flexes and coordinates with shoulder external rotation, causing the backward motion of the racket relative to the COM. This not only increases the swing distance but also pre-stretches the triceps. According to the Stretch-Shortening Cycle principle, the rapid stretching of muscles elicits a stretch reflex, which facilitates a higher velocity during the subsequent extension phase and provides optimal initial conditions for rapid elbow extension. This rapid extension is particularly crucial for maximizing racket head speed at impact, representing one of the significant factors positively influencing serve velocity(6, 27). Previous study indicated that 75.1% of the angular momentum is transferred to the racket through the motion of the trunk and upper extremity, with the largest proportions of angular momentum concentrated at the racket (35.9%) and the forearm (25.7%) (28). The elbow must complete extension in an extremely short period to ensure maximum energy transfer efficiency (3). Therefore, to increase the speed of elbow extension, it is essential to enhance the speed of elbow flexion during the cocking phase.

A notable technical difference between the two groups during the serve was in their trunk movement. The findings demonstrated that top-ranked players predominantly utilize trunk rotation, while lower-ranked players create racket lag through a composite trunk movement around the sagittal and frontal axes. Specifically, the ROM of trunk bending in lower-ranked players was more than twice that of their top-ranked counterparts—with the mean flexion and extension reaching 45° compared to 21° in top players, and lateral flexion averaging 43° vs. 18°. This excessive composite bending may result in the significantly slower serve velocity observed in the lower-ranked group (29). The essence of trunk movement is rotation around different axes of the body, with rotation around the vertical axis being more economical during the serve (30). The trunk rotation is the relative movement between shoulders and hips, primarily originating from shoulder movement during the serve (31). The radius of this rotation is approximately half of the shoulder width, which is less than that of trunk bending, resulting in a smaller moment of inertia. Furthermore, the shift in the COM during rotation is minimal, and the asymmetric load on the lumbar spine is reduced (22), which benefits movement stability. As the serve transitions from a side-facing stance to a forward-facing one, the movement manifests as rotation, transmitting the energy generated by the leg extension upwards through pelvic rotation. The trunk rotation follows this trend. The results suggest that lower-ranked players may be over-relying on exaggerated sagittal and frontal plane trunk movements to compensate for insufficient transverse-plane rotation. The excessive bending in lower-ranked players might also reflect an inability to decelerate the trunk.

The differences in trunk movements between the two groups likely arise from disparities in COM control and the specific details of lower extremity techniques. The findings indicated that at the MKF, the COM of lower-ranked players was positioned farther from the racket side foot—averaging 18.4 cm compared to 12.8 cm in top-ranked players—leading them to rely more on the non-racket side leg for support. A greater distance of COM to racket side foot at MKF correlates with lower ball velocity (32), a conclusion that is supported by the findings of this study. When the vertical axis of the body forms an angle with the vertical ground reaction force, the push-off movement may induce a tendency for the trunk to lean forward and sideways, and transferring the COM to the racket side leg helps to reduce this movement tendency. In terms of technique details of the lower extremity, this study found that the peak hip linear velocity of lower-ranked players occurred earlier than that of top-ranked players. At RLP, the server has minimal or even no contact with the ground (32). During the aerial phase, the hip undergoes slight flexion. On one hand, lower-ranked players may initiate hip flexion earlier; on the other hand, as they primarily create racket lag through trunk bending, it is speculated that they also commence pelvic rotation braking earlier. These two factors may contribute to the earlier occurrence of peak hip linear velocity.

This study has several limitations. First, due to constraints in data collection within competitive settings, the sample size was relatively small. Additionally, factors such as the mixed cross-season data, the psychological stress states of players in different match scenarios, racket string tension, and the types of balls used were not strictly controlled. These factors may affect the players’ competitive states, thereby influencing the ball velocity. Future research should consider expanding the sample size, incorporating more match data from players, and recording and controlling the aforementioned confounding variables as much as possible to enhance the rigor and generalizability of the conclusion. Second, this study analyzed only the fastest serve of each player in a single match, without considering the stability and consistency of the technical movements. Future research could collect and analyze multiple serves from players to evaluate their techniques more comprehensively. The current analysis focused on discrete kinematic parameters without evaluating coordination or kinetic chain efficiency. Integrating electromyography and kinetics would allow for a more thorough exploration of the key biomechanical mechanisms influencing in-match serve technique.

## 5 Conclusions

Top-ranked players generated higher serve velocity than lower-ranked players. Their serve kinematics were characterized by a more backward-leaning COM, a higher upper arm position, and a larger elbow angle at MKF. Additionally, top players exhibited a larger elbow angle at RLP and greater peak angular velocity of elbow flexion during the cocking phase, while demonstrating a smaller ROM in trunk bending during the acceleration phase. Coaches aiming to improve serve velocity should focus on specific technical adjustments. Specifically, players should be encouraged to maintain an elevated upper arm and an open elbow at MKF to optimize the subsequent acceleration space. Furthermore, emphasizing a rapid, relaxed racket drop and prioritizing trunk rotation over excessive trunk bending may help to improve serve velocity.

## Data Availability

The original contributions presented in the study are included in the article/Supplementary Material, further inquiries can be directed to the corresponding author.
