# Supplementary material for: Kinematic differences in the in-match serve between top-ranked and lower-ranked Women's Tennis Association players
Source: Front Sports Act Living. 2026 Apr 23;8:1787475. doi: 10.3389/fspor.2026.1787475 (PMC13149452; doi:10.3389/fspor.2026.1787475)
Supplement: Supplementary file 1 [file Supplementaryfile1.pdf]

1 Definition of segment and axis vector

In this study, joint angles were calculated using vector algebra within the global coordinate system. First, all anatomical landmarks were manually digitized frame-by-frame from each camera view (Table 1).

Table 1 Location descriptions of anatomical landmarks

	**Location description**
Head	Highest point of the mid-sagittal plane of the head.
Chin	Point on the mandible collinear with the Vertex and C7 markers.
Neck	Spinous process of the 7th cervical vertebra.
Xiphoid	Triangular depression inferior to the xiphoid process.
Navel	Center of the umbilicus.
Shoulder	The most prominent point of the middle deltoid.
Elbow	Midpoint of the elbow crease.
Wrist	Midpoint of the wrist crease.
Finger	3rd Metacarpophalangeal joint.
Hip	Anterior view: mid-inguinal point; Lateral view: greater trochanter.
Knee	Anterior view: center of the patella; Posterior view: popliteal fossa.
Ankle	Prominence of the malleolus.
Heel	Posterior center of the heel.
Toe	Anterior tip of the foot.

The longitudinal vectors of upper arm, forearm, hand, thigh, shank and foot were defined based on the coordinates of shoulder, elbow, wrist, finger, hip, knee, ankle and toe, as described in equations (1) to (6):

$$\overrightarrow{v_{upperarm}} = \overrightarrow{r_{elbow}} - \overrightarrow{r_{shoulder}} \tag{1}$$

$$\overrightarrow{v_{forearm}} = \overrightarrow{r_{wrist}} - \overrightarrow{r_{elbow}} \tag{2}$$

$$\overrightarrow{v_{hand}} = \overrightarrow{r_{finger}} - \overrightarrow{r_{wrist}} \tag{3}$$

$$\overrightarrow{v_{thigh}} = \overrightarrow{r_{knee}} - \overrightarrow{r_{hip}} \tag{4}$$

$$\overrightarrow{v_{shank}} = \overrightarrow{r_{ankle}} - \overrightarrow{r_{knee}} \tag{5}$$

$$\overrightarrow{v_{foot}} = \overrightarrow{r_{toe}} - \overrightarrow{r_{ankle}} \tag{6}$$

To calculate joint angle by segment planar projection, anatomical axes were established for the upper and lower segments. The unit vector of the frontal axis ($\hat{u}_F$) was defined as the vector pointing from the right joint to the left joint:

$$\hat{u}_F = \frac{\overrightarrow{r_L} - \overrightarrow{r_R}}{||\overrightarrow{r_L} - \overrightarrow{r_R}||} \tag{7}$$

where $\overrightarrow{r_L}$ and $\overrightarrow{r_R}$ are the coordinates of the left and right joint centers, respectively (shoulders for the upper axis; hips for the lower axis)

To define the sagittal axis, an auxiliary vector ($\overrightarrow{v_{aux}}$) was calculated connecting the center of the joints ($\overrightarrow{O_{mid}}$) to an auxiliary point ($\overrightarrow{r_i}$):

$$\overrightarrow{O_{mid}} = \frac{\overrightarrow{r_L} + \overrightarrow{r_R}}{2} \tag{8}$$

$$\overrightarrow{v_{aux}} = \overrightarrow{r_i} - \overrightarrow{O_{mid}} \tag{9}$$

The $\overrightarrow{r_i}$ used were the xiphoid for the upper and navel for the lower part.

The sagittal axis ($\hat{u}_S$) was defined as the cross product of the auxiliary vector and the frontal axis. To ensure the axis points anteriorly for both parts:

$$\hat{u}_S = \frac{\overrightarrow{v_{aux}} \times \hat{u}_F}{||\overrightarrow{v_{aux}} \times \overrightarrow{u_F}||} \text{ (upper frontal plane)} \tag{10}$$

$$\hat{u}_S = \frac{\hat{u}_F \times \overrightarrow{v_{aux}}}{||\overrightarrow{u_F} \times \overrightarrow{v_{aux}}||} \text{ (lower frontal plane)} \tag{11}$$

Finally, the point towards the ground vertical axis ($\hat{u}_V$) was calculated:

$$\hat{u}_V = \frac{\hat{u}_F \times \hat{u}_S}{||\hat{u}_F \times \hat{u}_S||} \tag{12}$$

2 Definition of joint angle

The projected angle (A_{proj}) was calculated based on the geometric relationship between the segment vector, the normal vector, and the reference axis:

$$\vec{n} \in \{\hat{u}_F, \hat{u}_S, \hat{u}_V\} \tag{13}$$

$$\overrightarrow{v_{proj}} = \overrightarrow{v_{seg}} - \left(\overrightarrow{v_{seg}} \cdot \vec{n}\right)\vec{n} \tag{14}$$

$$A_{proj} = cos^{-1}\left(\frac{\overrightarrow{v_{proj}} \cdot \overrightarrow{v_{ref}}}{\left\|\overrightarrow{v_{proj}}\right\|\left\|\overrightarrow{v_{ref}}\right\|}\right) \tag{15}$$

$\vec{n}$ is the unit vector defining the anatomical plane of interest. And $\overrightarrow{v_{ref}}$ is the axis lying within the plane, representing 0° reference line for the angle calculation. $\overrightarrow{v_{proj}}$ represents the orthogonal projection of the segment vector on to the specified plane.

Based on the definition above, the upper extremity joint angles were defined based on the projections of segment vectors onto the upper planes (Figure 1):

- Shoulder flexion/extension: the angle between the projection of the upper arm vector onto the sagittal plane and vertical axis.
- Shoulder adduction/abduction: the angle between the projection of the upper arm vector onto the frontal plane and vertical axis.
- Shoulder horizontal adduction/abduction: the angle between the projection of the upper arm vector onto the transverse plane and frontal axis.
- Shoulder external/internal rotation: the angle between the projection of the forearm vector and the sagittal axis onto the transverse plane of the upper arm (the plane perpendicular to the upper arm vector).
- Elbow angle: the supplementary angle to the angle between the upper arm and the forearm vector.
- Wrist angle: due to the restrictions on attaching additional markers to the players' wrists during competition, the wrist joint motion was approximated. Wrist angle was defined as the angle between the forearm and the hand vector.

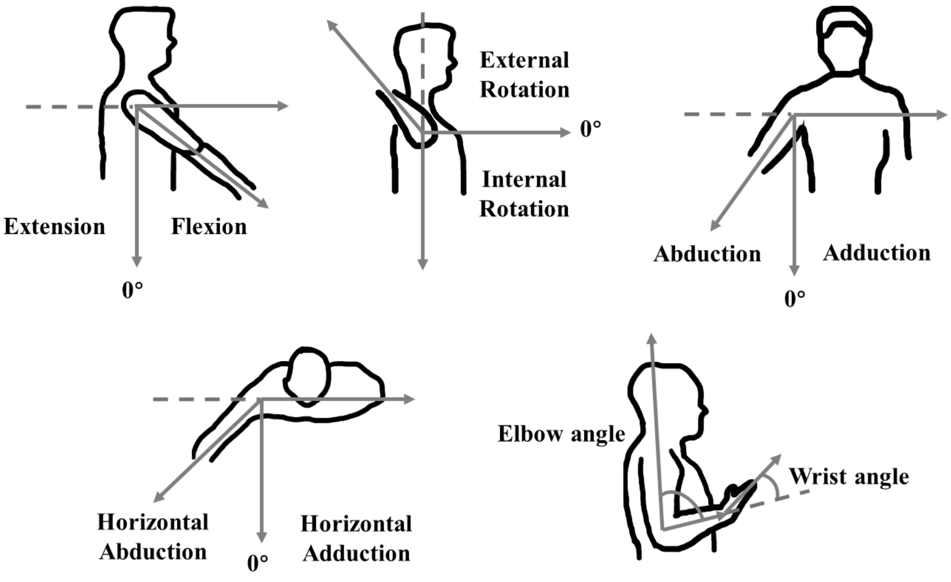

Figure 1 Upper extremity joint angle definitions.

The lower extremity joint angles were defined based on the projections of segment vectors onto the lower planes (Figure 2):

- Hip flexion/extension: the angle between the projection of the thigh vector onto the sagittal plane and the vertical axis.
- Knee angle: the supplementary angle to the angle between the thigh and the shank vector.
- Ankle angle: the angle between the projection of the shank and the foot vector.

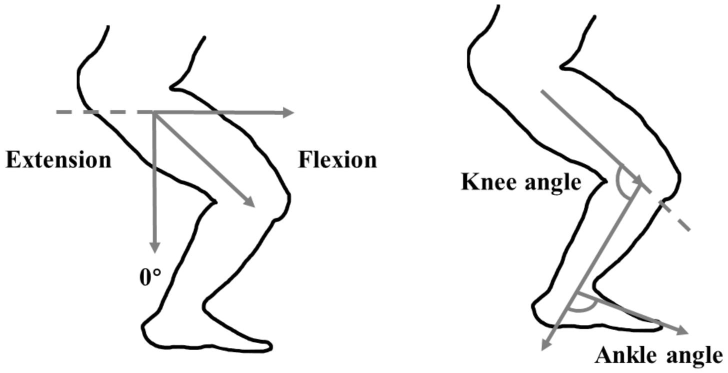

Figure 2 Lower extremity joint angle definitions.

Trunk angles were defined by projecting the upper trunk segment axes onto the lower planes:

- Trunk axial rotation: the angle between the projections of the upper frontal axis and the lower frontal axis onto the transverse plane.
- Trunk lateral flexion: the angle between the projections of the upper vertical axis and the lower vertical axis onto the frontal plane.
- Trunk flexion/extension: the angle between the projections of the upper sagittal axis and the lower sagittal axis onto the sagittal plane.
